# Identification of Neural Outgrowth Genes using Genome-Wide RNAi

**DOI:** 10.1371/journal.pgen.1000111

**Published:** 2008-07-04

**Authors:** Katharine J. Sepp, Pengyu Hong, Sofia B. Lizarraga, Judy S. Liu, Luis A. Mejia, Christopher A. Walsh, Norbert Perrimon

**Affiliations:** 1 Department of Genetics, Harvard Medical School, Boston, Massachusetts, United States of America; 2 Department of Computer Science, National Center for Behavioral Genomics, Brandeis University, Waltham, Massachusetts, United States of America; 3 Howard Hughes Medical Institute, Beth Israel Deaconess Medical Center, Division of Genetics, Children's Hospital, Harvard Medical School, Boston, Massachusetts, United States of America; 4 Department of Neurology, Harvard Medical School, Boston, Massachusetts, United States of America; 5 Howard Hughes Medical Institute, Harvard Medical School, Boston, Massachusetts, United States of America; Northwestern University, Howard Hughes Medical Institute, United States of America

## Abstract

While genetic screens have identified many genes essential for neurite outgrowth, they have been limited in their ability to identify neural genes that also have earlier critical roles in the gastrula, or neural genes for which maternally contributed RNA compensates for gene mutations in the zygote. To address this, we developed methods to screen the *Drosophila* genome using RNA-interference (RNAi) on primary neural cells and present the results of the first full-genome RNAi screen in neurons. We used live-cell imaging and quantitative image analysis to characterize the morphological phenotypes of fluorescently labelled primary neurons and glia in response to RNAi-mediated gene knockdown. From the full genome screen, we focused our analysis on 104 evolutionarily conserved genes that when downregulated by RNAi, have morphological defects such as reduced axon extension, excessive branching, loss of fasciculation, and blebbing. To assist in the phenotypic analysis of the large data sets, we generated image analysis algorithms that could assess the statistical significance of the mutant phenotypes. The algorithms were essential for the analysis of the thousands of images generated by the screening process and will become a valuable tool for future genome-wide screens in primary neurons. Our analysis revealed unexpected, essential roles in neurite outgrowth for genes representing a wide range of functional categories including signalling molecules, enzymes, channels, receptors, and cytoskeletal proteins. We also found that genes known to be involved in protein and vesicle trafficking showed similar RNAi phenotypes. We confirmed phenotypes of the protein trafficking genes *Sec61alpha* and *Ran GTPase* using *Drosophila* embryo and mouse embryonic cerebral cortical neurons, respectively. Collectively, our results showed that RNAi phenotypes in primary neural culture can parallel *in vivo* phenotypes, and the screening technique can be used to identify many new genes that have important functions in the nervous system.

## Author Summary

Development and function of the brain requires the coordinated action of thousands of genes, and currently we understand the roles of only a small fraction of them. Recent advances in genomics, such as the sequencing of entire genomes and the discovery of RNA-interference as a means of testing the effects of gene loss, have opened up the possibility to systematically analyze the function of all known and predicted genes in an organism. Until now, this type of functional genomics approach has not been applied to the study of very complex cells, such as the brain's neurons, on a full-genome scale. In this work, we developed techniques to test all genes, one by one in a rapid manner, for their potential role in neuronal development using neurons isolated from fruit fly embryos. These results yielded a global perspective of what types of genes are necessary for brain development; importantly, they show that a large variety of genes can be studied in this way.

## Introduction

Many genes required for neurodevelopment have been identified as a result of large-scale genetic screening in *Drosophila*. It has been a choice model in neurogenetics since it has significant gene homology and anatomical similarity to vertebrates and the tools available for genetic manipulation are advanced. Recently, RNA-interference (RNAi) has provided an important new tool for genetic analysis since it can efficiently knock down gene expression within the *Drosophila* nervous system to replicate genetic hypomorphic and null mutant phenotypes [1],[2]. In *Drosophila*, RNAi is mediated by the introduction of long double stranded RNAs (dsRNAs). The internalized dsRNAs are processed into 21- to 23-nucleotide segments by Dicer and are incorporated into RISC protein complexes for degradation of mRNA transcripts [3],[4]. In simple model organisms such as *C. elegans* and *Drosophila*, RNAi analysis has been applied to the entire genome [5]. In addition, the use of cell-based RNAi assays enable genome-wide screens to be carried out in a high-throughput manner [6]. However, these high-throughput screening methods have been inaccessible to address questions in neurons due to the lack of appropriate cell lines that retain neuronal morphology, gene expression profiles, and electrophysiological activity. Thus to carry out a full genome analysis of neural development, we have adapted methods for RNAi screening by using *Drosophila* primary neural cultures and live-cell imaging.

A major advantage of using RNAi on primary cultures is that pleiotropic genes can be identified. Most genes have complex expression profiles that are not restricted to a single tissue type, and that can also include mRNAs that are maternally deposited into the egg. The primary cell culture method we present here isolates wild-type neuroblasts that are subsequently treated with RNAi, thus secondary cell defects due to disruption of tissues that form prior to neurogenesis can be avoided. In this way, primary neural culture RNAi could offer great potential to identify interesting novel genes that would be much more difficult to find using traditional screening methods.

Here we present the results from the first genome-wide RNAi analysis of live, fluorescently labeled primary neural cells for their effects on neural outgrowth and morphology. Through successive rounds of experimental replication, we identified 104 evolutionarily conserved genes that we implicate in neural development and function. For the phenotypic analysis, we developed computational image analysis methods that quantify specific morphological features of the cells. The statistical analysis can aid in the prediction of gene functions based on comparison of RNAi-induced phenotypic profiles of unknown genes to profiles that represent genes with known functions. To explore whether *in vitro* RNAi phenotypes from the genome-wide screen show analogous phenotypes *in vivo* and across species, we chose two genes involved in the protein trafficking category for further analysis. We found that *Sec61α* and *Ran GTPase* showed similar phenotypes in *Drosophila* embryos and embryonic mouse cortical neuron explants respectively. Both genes have complex expression patterns, and are important genes in human neurological disease pathways [7],[8]. The work thus demonstrates the advantages of using full-genome RNAi in *Drosophila* primary neural cells as a tool to gain novel insights into gene functions in the nervous system.

## Results

### Generation of Primary Neurons for RNAi

The characteristics of primary neuronal cultures generated from gastrula stage *Drosophila* embryos have been well characterized and their development mirrors the phases of proliferation, differentiation, outgrowth and mature physiological function in the intact organism. Primary neurons develop from neuroblasts that give rise to clusters of approximately 16 daughter cells [9],[10]. The neuronal clusters form miniature ganglia organized with neuronal cell bodies surrounding a central neuropil that contains high levels of presynaptic proteins [11]–[13]. Primary neurons can be either uni- or multipolar, and they extend microtubule-rich axons out of the cell clusters using growth cones bearing filopodia and lamellipodia [13]–[16]. The axons terminate on neighboring ganglia and contractile muscle fibers to form neural networks [11],[17]. In addition, glial cells either remain within the ganglia or extend along and wrap the axonal projections [10],[13],[18]. Axons terminating on muscles develop neuromuscular junctions, which show ultrastructural specializations typical of synapses, including synaptic vesicles and electron dense material bridging regions of the synaptic cleft [10]. The synapses of the primary neurons are electrophysiologically active and Na+-driven action potentials can be blocked by tetrodotoxin [10],[13],[19],[20].

The composition of neuronal types within primary cultures derived from gastrula stage embryos has been determined using immunostaining of molecular markers. Primary *Drosophila* neurons are Elav- and HRP-positive, and the subsets of motor and sensory neurons express Fasciclin II and Futsch respectively [13],[16]. Other markers, such as Neuroglian, Even-skipped are expressed by primary neurons, and glia are Repo-positive [13],[16]. Together, the morphological, physiological, and molecular characterizations demonstrate that primary neurons and glia retain a great number of characteristics from the *in vivo* situation, and thus make a very appropriate system for high-throughput functional genomics applications such as RNAi.

To carry out a full-genome RNAi screen for neural outgrowth and morphology, we developed methods to generate large-scale cultures of green fluorescent protein (GFP) -labeled primary neurons and glia from gastrula stage embryos. In addition, RNAi techniques were modified for higher efficacy on primary cells within a 384-well plate screening format. It has been demonstrated that RNAi knockdowns can replicate neuronal mutant phenotypes in *Drosophila* embryos [21],[22], and that RNAi can effectively knock down specific genes in vertebrate cultured neurons [23]. We also demonstrated that RNAi can significantly knock down gene expression in *Drosophila* primary neurons. For example, cultures treated with Neuroglian dsRNA had greatly reduced immunolabeling with anti-Neuroglian as compared to controls (Figure S1). In addition, we demonstrated that the RNAi assay can produce repeatable phenotypes within the networks of dissociated cells using positive controls that were expected to affect outgrowth in neurons. For example, by knocking down the cytoskeletal proteins Actin and beta-Tubulin, we demonstrated that the RNAi outgrowth phenotypes were consistent, robust, and stereotyped amongst independent cell culture preparations and amongst repeated wells within the same culture preparation (Figure S2A–C). Importantly, the phenotype characteristics were distinctive depending on which gene was targeted for knockdown, with sinuous axon trajectories in *Actin* knockdowns versus markedly reduced axon lengths in the *beta-Tubulin* RNAi cultures. Thus RNAi-induced phenotypes can result in significant visible morphological defects that are reproducible in primary neural cells.

**Figure 1:**
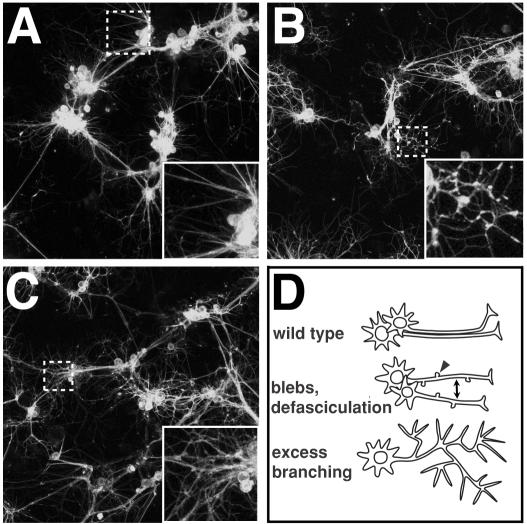
Morphological phenotype features and gene classes observed in RNAi screen. (A–C) Confocal micrographs of live, GFP-labeled neurons and glia. Inset on bottom right hand corner is enlargement of dotted small box areas in same image. (A) Wild-type (no dsRNA) control culture shows well fasciculated cell processes connecting cell body clusters. (B) Hydrogen-transporting ATPase *VhaAC39* dsRNA treated cells show increased varicosities (blebs) along cell processes, and excessive branching relative to controls. (C) Novel gene *CG14883* dsRNA treated cells show a combination of excessive branching and defasciculation. (D) Cartoon of axon morphologies in wild-type, and RNAi-treated cells. Wild-type axons emanating from neurons in the same cell body cluster often extend together in bundles (top). Axonal blebs are indicated with arrowhead and lack of bundling (defasciculation) is indicated by double-ended arrow.

For screening assay optimization, we performed pilot tests using a small collection of dsRNAs. Wild-type and negative control cultures had a stereotypic morphology, characterized by cell body clusters interconnected by well-fasciculated axonal tracts (Figure 1A). In contrast, a selection of the pilot test dsRNAs caused a variety of morphological defects associated with axonal tracts. For example, dsRNAs for hydrogen-transporting ATPase *VhaAC39* and novel gene *CG14883* caused excessive branching, blebbing, and defasciculation as well as disruption of cell cluster sizes (Figure 1B–D). Our observations supported previous analyses of primary neurons which showed that healthy cultured *Drosophila* neurons are well-fasciculated, while neurons disrupted by mutation or chemical treatments show branching abnormality, reduced axon lengths, and varicosities [13],[14],[16],[17].

### Full-Genome RNAi Screen in Primary Neural Cells

For the genome-wide screen, we used a library containing ∼21,300 dsRNAs, representing approximately 99% of annotated genes as well as additional predicted genes [24],[25]. Primary cell culture preparations were applied to 384-well plates pre-aliquoted with dsRNAs and incubated for one week (Figure S2D). The morphologies of the living GFP-labeled cells in response to RNAi were imaged on a robotic microscope and also visually scored. From visual inspection, morphological phenotypes were scored for excessive branching, defasciculation, axon blebbing, cell loss, and reduced outgrowth.

The initial full genome screen identified 336 dsRNAs that caused strong phenotypes and a further 2,106 dsRNAs that generated moderate to weak phenotypes. The identified genes encompassed most functional categories, with a large proportion being novel genes with no predicted function. In the initial screen, 136 dsRNAs that resulted in visible phenotypes represented genes that were previously shown to be required in *Drosophila* neurons or glia (Table S1). These functionally diverse genes included *Notch* (determination), *MAPK/rolled* (signaling), *Insulin-like Receptor* (growth and differentiation), *MAP1B/Futsch* (microtubule binding), *Rac2* (Actin dynamics), *Frizzled* (signal transduction), *Synaptobrevin* (synaptic vesicle release), *Kinesin light-chain* (axon transport), and *V-gated K^+^ channel/ether a go-go* (electophysiological signaling).

### Validation of *in vitro* RNAi Phenotypes

From the full-genome screen, 125 genes were selected for confirmation of the RNAi primary cell phenotypes. These included all the candidates that had a strongly penetrant RNAi phenotype and that have vertebrate homology using Reciprocal-Best-Blast and other criteria. Proteasomal and ribosomal genes were excluded due to their widespread cellular functions. The dsRNAs for the 125 candidates used in the full genome screen were resynthesized for multiple replicate analysis. To control for potential off-target effects caused by long dsRNAs [26],[27], for each gene, 1–2 additional non-overlapping dsRNAs that had no homology to other genes at a statistically determined 17- to 19-basepair cut-off threshold [26],[27] were synthesized. The primary culture and RNAi conditions used in the full genome screen were carried out blindly on 12 replicates. With the secondary screen, the phenotypes of 104 of the initial 125 selected hits were confirmed with 2 or more independent dsRNAs (Table 1).

**Table 1:** Genes implicated in neural development. All listed genes show disrupted primary neuronal phenotypes with 2 or more independent RNAi amplicons.

Drosophila Gene ID	Drosophila Gene Name	Human Homology	Mouse Homology	Description
**Channels**				
CG4370	Irk2	KCNJ9	Kcnj9	inward rectifier potassium channel
CG16793		PKD1L2	Pkd1l2	Ca2+ channel
CG12904		KCNT1	C030030G16Rik	Ca2+-activated K+ channel
CG1066	Shaker cognate b	KCNB2	Kcnb2	V-gated K+ channel
**Transporters**				
CG18660	Nckx30C	SLC24A2	Slc24a2	Ca2+, K+∶Na+ antiporter
CG10413		SLC12A9	Slc12a9	Na+∶K+∶Cl− symporter
**Receptors**				
CG31092	LpR2	VLDLR	Vldlr	low-density lipoprotein receptor
CG11155		GRIK4	Grik4	kainate glutamate receptor
CG4875		RCP9	Crcp	G-protein coupled receptor
CG7535	GluClalpha	GLRA3	Glra3	GABA receptor activity
CG5911	ETHR	GHSR	Ghsr	neuropeptide receptor
**Cell Adhesion**				
CG1634	Nrg	L1CAM	C130076O07Rik	Ig cell adhesion
CG14762		GP5	Gp5	LRR cell adhesion
CG16974		LRRN5	Lrrn2	LRR cell adhesion
**Cytoskeleton Associated**				
CG32137		LOC92558	BC038613	microtubule binding
CG17461	Kif3C	KIF17	Kif17	microtubule motor
CG6450	lava lamp	CEP2	Cep2	cytoskeleton binding
CG10642	Klp64D	KIF3A	Kif3a	kinesin microtubule motor
CG32138		FMNL2	Fmnl2	actin binding
CG3299	Vinculin	VCL	Vinculin	actin binding
CG1200	Aplip1	MAPK8IP1	Mapk8ip1	kinesin binding
CG6224	diablo	KLHL20	Klhl20	actin binding
CG14535		FLJ10157		microtubule binding
CG9995	Huntingtin	HD		microtubule binding
**Vesicle and Protein Transport**				
CG7961	alphaCop	COPA	Copa	protein transport
CG9539	Sec61alpha	SEC61A2	Sec61a2	protein transport
CG1250	sec23	SEC23A	Sec23a	exocytosis
CG10130	Sec61beta	SEC61B	Sec61b	protein transport
CG7740	prominin-like	PROM1	Prom1	protein transport
CG6095		EXOC8	Exoc8	exocyst component
CG6625	Snap	NAPA	Napa	soluble NSF attachment
CG1528	gammaCop	COPG	Copg	protein transport
CG9778		SYT14	Syt14	vesicle tranpsort
CG7360	Nup58	NUPL1	Nupl1	nucleocytoplasmic transport
**Transcription**				
CG13316	Mnt	MAD	Mxd3	RNA pol II transcription factor
CG4539	Bekka	C14orf111	LOC269546	transcriptional activator
CG3871	Six4	SIX4		RNA pol II transcription factor
CG31256	Brf	BRF1	Brf1	transcription factor binding
CG9305		BDP1	G630013P12Rik	transcription factor
CG5890		CSEN	Csen	presenilin binding, transcription
**DNA/RNA associated**				
CG3658	CDC45L	CDC45L	Cdc45l	DNA binding
CG10327	TBPH	TARDBP	Tardbp	mRNA binding
CG12352	separation anxiety	MAK3	Mak3	mitotic chromatid adhesion
CG7187	Ssdp	SSBP3	Ssbp3	single stranded DNA binding
CG40411	Parp	PARP1	Parp1	DNA binding/repair
CG7154		BRD7	Brd7	DNA binding
CG9677	Int6	EIF3S6	Eif3s6	translation initiation factor
CG18009	Trf2	TBPL1	Tbpl1	DNA binding
CG9633	RpA-70	RPA1	Rpa1	single stranded DNA binding
CG10215	Ercc1	ERCC1	Ercc1	damaged DNA binding
CG13298		SF3B14	0610009D07Rik	mRNA splicing factor
CG4236	Caf1	RBBP4	Rbbp4	histone binding
**Enzymes**				
CG1970		NDUFS2	Ndufs2	NADH dehydrogenase
CG12082		USP5	Usp5	ubiquitin specific protease
CG10679	Nedd8	NEDD8	Nedd8	protein catabolism
CG2656		MGC14560	D5Ertd708e	ATP binding
CG8891		ITPA	Itpa	nucleic acid metabolism
CG32056		PLSCR1	LOC433328	phospholipid scramblase
CG9372		KLKB1	Klkb1	protease
CG12077		PIGC	Pigc	glycosyl transferase
CG7266	Eip71CD	MSRA	Msra	methionine sulfoxide reductase
CG4829		GGT1	Ggt1	acyltransferase
CG31871		LIPL1	Lip1	triacylglycerol lipase
CG4842		HPGD	Hpgd	oxidoreductase
CG33085		ASL	Asl	argininosuccinate lyase
**Signal Transduction**				
CG10257		FAIM	Faim	apoptotic inhibitory protein
CG1954	Pkc98E	PRKCE	Prkce	protein kinase C
CG1676	cactin	C19orf29		signal transduction
CG18803	Presenilin	PSEN2	Psen2	receptor peptidase
CG9738	Mkk4	MAP2K4	Map2k4	MAP kinase kinase
CG9098		BCAR3	Bcar3	SH2/SH3 adaptor
CG31349	polychaetoid	TJP1	Tjp1	guanylate kinase
CG1848	LIMK1	LIMK1	Limk1	protein kinase
CG31475		Cab45	Sdf4	calcium mediated signaling
CG18247	shark	ZAP70	Zap70	protein kinase
CG30021	skiff	MPP7	Mpp7	guanylate kinase
CG14782		PLEKHF2	Plekhf2	guanyl-nucleotide exchange factor
CG1404	ran	RAN	Ran	GTP binding
CG7156		RPS6KC1	Rps6kc1	protein kinase
CG9222		TSSK4	Tssk4	protein kinase
CG10951	niki	NEK8	Nek8	protein kinase
**Novel Genes**				
CG1109		WDR33	WD REPEAT 33	
CG7146		VPS39	Vps39	
CG5484		YIF1B	Yif1b	
CG3305		LAMP1		
CG3403		PREI3	Prei3	
CG18675		C21orf59	1110004E09Rik	
CG2691		KIAA0690	AA408556	
CG4893		CGI-38	2700055K07Rik	
CG31126		CGI-143	1810037G04Rik	
CG13990			Gm256	
CG14883		MIR16	Mir16	
CG8309		GA17	Ga17	
CG1463		FLJ20729	2410019A14Rik	
CG31917		ZNHIT1	Znhit1	
CG3703		RUNDC1	Rundc1	
CG5642		EIF3S6IP	Eif3s6ip	
CG32685			Ylpm1	
CG12078		HSPC129	D2Ertd485e	
CG10059	MAGE	MAGED1	Ndnl2	
CG6145		FLJ13052	BC004012	
CG10249		ANKRD15	D17Ertd288e	
CG8055		CHMP4B	2010012F05Rik	
CG11448		FLJ39378	2900002H16Rik	

**Figure 2:**
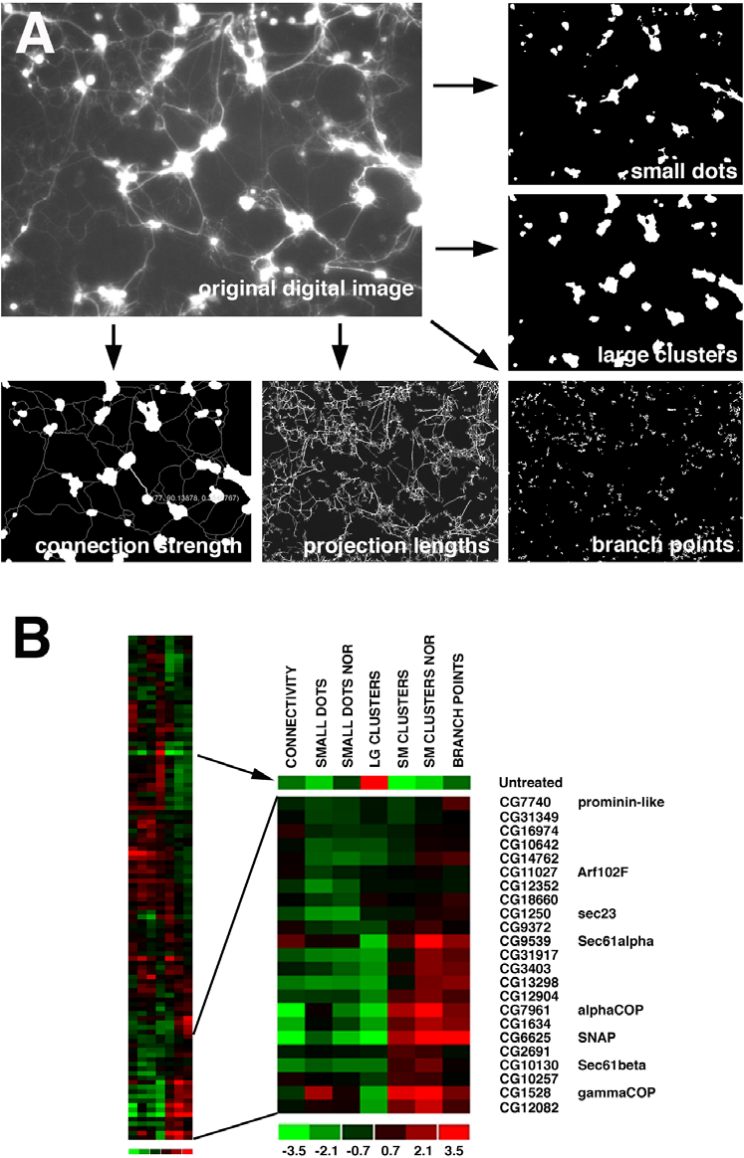
Quantification of RNAi phenotypes and cluster analysis. (A) Screen images acquired on a robotic widefield fluorescence microscope were analyzed by custom image analysis software. Image features relating to the health of the cultures were extracted using filters that measured the amount of small dots (indicator of debris and blebbing), small, medium and large cell clusters, lengths of processes and numbers of branch points, and the strength of connectivity between large cell clusters. Indicators of weaker primary neural health are decreased sizes of cell clusters, increased branch points, decreased projection lengths, and decreased interconnectivity between cell clusters. (B) For each image feature quantified in (A), heat map representation values were assigned to each of the quantified features, where increased values are in red and decreased values are in green. All the quantified image features for each gene were assembled into a single profile and the profiles were ordered into a hierarchical cluster as shown. Normalized (NOR) feature quantifications are included. Protein and vesicle trafficking genes were largely located to the bottom region of the hierarchy as shown in highlighted region.

To statistically analyze the morphological characteristics of the GFP-labeled cells imaged on the robotic microscope, digital image analysis tools were developed, since existing commercial image analysis software packages were far too generic for the analysis of the *Drosophila* neural cultures. Image features, including the amount of branched regions, sizes of cell clusters, lengths of fascicles, and degrees of connectivity were quantified (Figure 2A) and normalized against controls within the replicate plates. Using the Multivariate Student's t-test with a statistical cutoff of p≤0.001, 83% of the genes retested showed RNAi phenotypes significantly different than wild-type controls with two or more independent dsRNAs (Table 1). Genes that retested as significantly different from wild-type controls with only one dsRNA are reported in Table S2. For the quantified image features, heat-map hierarchical clusters were generated (Figure 2B, Figure S3). For the heat map, the quantified values of the image features were represented by a color code (red and green, Figure 2B) where red values show an increase in the phenotype value and green shades show decreases in the phenotype value according to the designated scale. For example, *SNAP* RNAi caused disruption of cell proliferation that would normally generate healthy, large sized cell clusters. Thus there was an overall increase in the number of small cell clusters, as represented by the red shading of the “small clusters” category. Interestingly, by using blebbing, connectivity, and branching features for cluster analysis, we found that genes associated with vesicle and protein trafficking were localized to a similar region within the hierarchy (Figure 2B).

Hierarchical cluster analysis could assist in the prediction of roles for genes with no known functional motifs, since genes of similar function are likely to have a greater chance of localizing to similar regions of the hierarchy. For example, the novel gene *CG3403* was mapped in the highlighted cluster region (Figure 2B). The rat homolog of *CG3403* is *Phocein*, and it is reported to localize to the neuronal Golgi apparatus and dendritic spines [28]. Based on its sequence similarity to clathrin adaptor proteins, it is hypothesized to be involved in vesicular endocytosis, however its precise function is unknown. Importantly, growth cone dynamics are mediated by endocytosis in a similar manner to vesicle recycling at the synapse [29]. Thus it is possible from the RNAi outgrowth phenotypes and clustering analysis that *CG3403* could be involved also in protein trafficking during axon outgrowth.

General bioinformatics tools were also informative for analysis of the candidate genes. For example, from current database information, at least 55% of the validated genes have expression within nervous system tissue during *Drosophila* embryogenesis. A significant portion of the genes with embryonic neural expression also had maternally deposited mRNA for the same gene in the early embryo such as *Caf1*, *Lpr2*, and *MAGE*. It is possible that many of these genes have not been detected in previous screens for embryonic nervous system patterning, because maternally deposited mRNAs can sometimes compensate for the loss of zygotically expressed transcripts during early development.

### Phenotypes and Functions of Selected Hits

**Figure 3:**
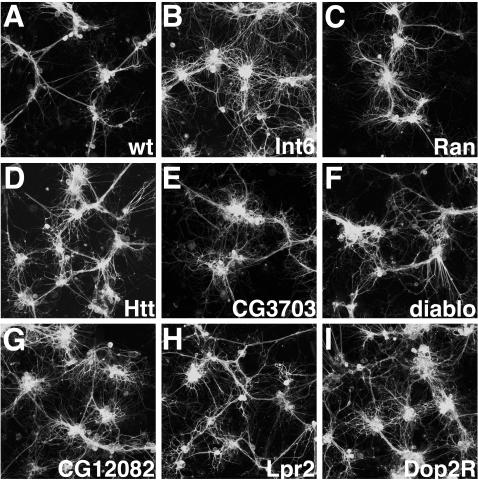
Confocal micrographs of live GFP-labeled primary neural cultures treated with dsRNAs showing altered cell morphologies. All cultures were grown on glass coverslips coated with poly-L-lysine. (A) Wild type (negative control). Wild type primary neurons in a mature culture show cell body clusters interconnected by well-fasciculated axon tracts. (B) *Int6* transcription initiation factor knockdowns show extensive defasciculation. (C) *Ran* GTPase RNAi cultures have both excessive branching and defasciculation. (D) *Huntingtin* knockdowns show a moderate level of excessive branching. (E) *Sec61α* RNAi shows poor connectivity between cell clusters and highly branched, defasciculated neurons. (F) *Diablo* (cytoskeletal binding protein). *Diablo* knockdown leads to a primarily defasciculated phenotype. (G) *CG12082* (novel gene) RNAi causes reduced connectivity between cel clusters, excessive branching and defasciculation. (H) *Lpr2* LDL receptor knockdowns show excessive branching and defasciculation, yet with robust outgrowth. (I) *Dopamine 2-like Receptor* RNAi shows defasciculation.

A selection of the validated genes was morphologically analyzed in greater detail using confocal microscopy and pan-neural markers (Figure 3). Relative to control cultures (Figure 1A, 3A), reduced fasciculation and increased branching of processes were the most widespread phenotypes observed. While these two attributes were often observed in combination, many dsRNAs generated distinctive phenotypes. For example, knockdowns of the translation initiation factor *Int6* and *ran GTPase* both caused excessive branching and defasciculation, however their morphological profiles were quite different due to the relative amounts of each feature (Figure 3B,C). Further knockdowns of gene expression including: *Huntingtin*, *Sec61α*, actin binding gene *diablo*, novel gene *CG12082*, LDL receptor *Lpr2*, and *Dopamine 2-like Receptor* (*CG9569*), also showed distinctive characteristics of morphological disruption (Figure 3D–I). Given the diversity of complex phenotypes of neural morphology in cell culture, the importance of using computer algorithms to quantify specific features is underscored. Thus image analysis algorithms such as those presented here will be useful in future suppressor/enhancer screens or chemical screens in primary neural cells.

From the RNAi phenotypes examined in greater detail, the *Dopamine 2-like Receptor* (Figure 3I) was of interest and showed an excessive branching phenotype. Although neurotransmitter receptors are most widely known for their central role in synaptic transmission, they have also been implicated in axon outgrowth [30] and are expressed during early neurodevelopment, prior to the establishment of synapses [31]–[33]. Interestingly, *dopa decarboxylase* deficiency mutants in *Drosophila*, which are unable to synthesize serotonin and dopamine, show an extensive increase in axonal branching in the larva [34].

Ion channels were also represented amongst the validated RNAi candidates. For example, we found that knockdown of *CG16793*, a calcium channel, showed disruption of neuronal growth in culture, with increases of branch points and weakened connectivity of axons between neighboring cell body clusters (Figure S3). During axonal outgrowth, calcium transients are important in regulating the advance of growth cones [35] and it has been demonstrated in cortical neurons that voltage-gated calcium channels mediate this activity [36]. Increased calcium transients result in slowed growth cone advancement [36],[37]. Changes in the migration rates and sizes of growth cones during outgrowth are correlated with the demarcation of axonal branch points and it is now hypothesized that the local changes in calcium activity could ultimately lead to the activation local branching morphogenesis [36]. It has also been observed that the neurotransmitter serotonin can enhance neurite outgrowth through the activation of serotonin receptors and voltage gated calcium channels [38]. Thus our observations support the findings that altered calcium signaling can lead to changes in axon outgrowth and branch patterning.

The screen identified numerous DNA- and RNA-associated genes that have poorly understood roles in the nervous system. It is thought that during neurodevelopment, postmitotic neurons are highly vulnerable to DNA damage [39]. Thus genes involved in DNA repair have an influence on the genesis of the nervous system. In our screen we identified *Parp*, the *Drosophila* homolog of *PARP-1*, which has a well-characterized role in DNA repair [40]. Our data support recent work in rat cortical neurons suggesting that PARP-1 may also have a neurotrophic role [41].

Genes relevant to neuropathological disorders were also identified in the screen, including *Presenilin*, *Huntingtin*, and *Prominin-like*. The human orthologs of these genes are implicated in Alzheimer's, Huntington's, and retinal degeneration diseases respectively. The RNAi phenotype of *Huntingtin* shows increased branching and defasciculation (Figure 3D), and suggests that the wild type form of *Huntingtin* is important for proper nervous system function. This observation is in agreement with previous observations on Huntingtin loss [42]. In Huntington's disease, Huntingtin protein is thought to acquire gain of function due to expanded polyglutamine repeats. Genetic modifier screens using *in vivo* Huntington's disease models in flies have been successful in identifying evolutionarily conserved suppressors of polyglutamine expansion [43]. Potentially, by using primary cell based RNAi, this type of screen could be carried out on the entire genome in a high-throughput manner, and thus identify potential new drug targets for the development of therapeutics.

### Analysis of *Sec61α in vivo*

The screen identified a significant number of genes involved in vesicle and protein trafficking including *SNAP*, *sec23*, *αCOP*, *γCOP*, *Ran GTPase*, *Arf102F*, and both *Sec61α* and *Sec61β*. Protein trafficking is most likely a key process in generating the highly polarized structure of neurons. Yet this class of genes is difficult to study in a neuronal context due to their widespread expression. Thus the use of RNAi on dissociated neurons could also yield new insights into the functions of these genes.

**Figure 4:**
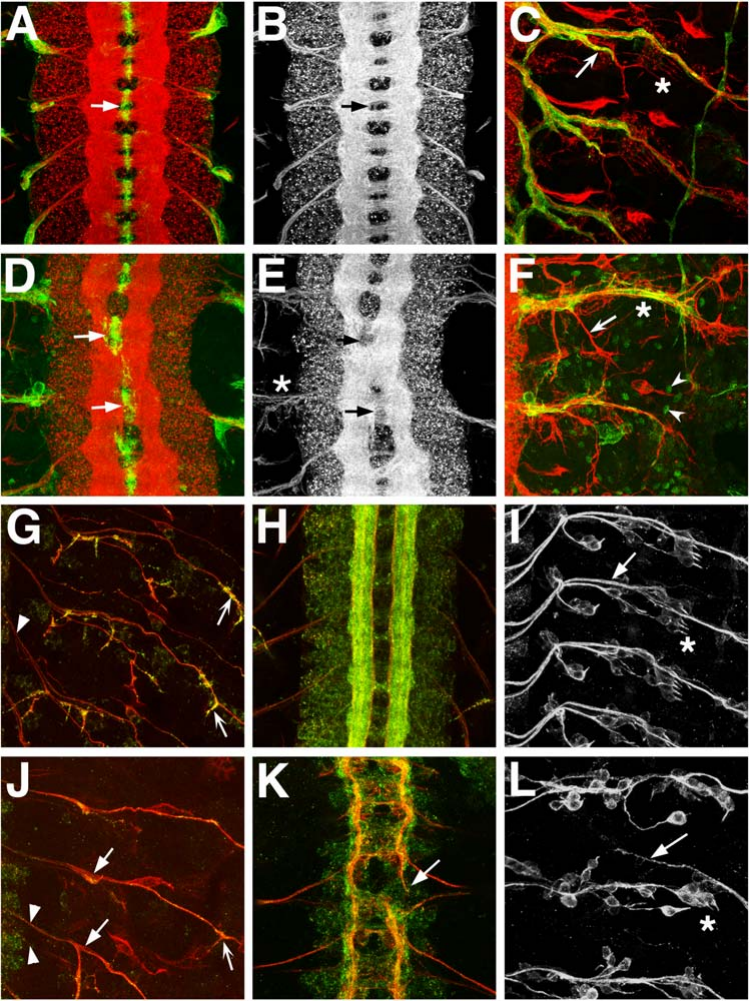
Phenotypes of wild type and Sec61α mutant embryonic nervous systems. (A–C, G–I) Wild type. (D–F) *Sec61α^SH190^* homozygous mutant. (J–L) *Sec61α^k04917^* homozygous mutant. Anterior is to the top. PNS images have CNS off to the left in C,F,G,I,J,L. (A–C) Neurons are labeled with anti-HRP (red) and glia are labeled with mAb 5B12 (green). (G,H,J,K) Motor neurons labeled with mAb 1D4 (red) and anti-Synaptotagmin-1 (green). (I,L) Sensory neurons labeled with mAb 22C10. All embryos are late embryonic stage, approximately 22 hours old. (A) Wild-type CNS shows ladder-like pattern of axon tracts with commissural tracts ensheathed by midline glia (arrow). (B) Red channel (neurons) only of (A). (C) Wild-type PNS shows stereotypic pattern of motor- and sensory neuron projections (red) and coverage of nerve tracts by peripheral glia (green). SNa branch is indicated with arrow. (D) *Sec61α* embryos show disruption of CNS axon tract pattern (see also E), with glial profiles displaced compared to wild type (arrows). The confocal laser power relative to that used for imaging the wild type was increased to show glial staining pattern. (E) Neurons from (D) shown, highlighting lack of separation of central commissures (arrows). Defasciculation of peripheral axon projections at the CNS/PNS transition zone (asterisk) is also evident. (F) *Sec61α* mutant PNS shows variable disruption of axon patterning across hemisegments. Poor glial coverage of mistargeted SNa branch is indicated (arrow). Ectopic expression of mAb5B12 antigen is observed in the periphery (arrowheads). (G) Wild type PNS motor neuron pattern (red). Peripheral nerves near CNS/PNS transition zone are well fasciculated and tightly bundled (arrowhead). Synaptotagmin-1 is strongly detected at motor axon termini (arrows). (H) Wild type CNS motor neuron pattern (red) and distribution of synapse marker Synaptotagmin-1 (green). (I) Wild type PNS sensory neuron pattern. Arrow shows Anterior Fascicle sensory neuron tract pathway leading towards CNS on the left. (J) *Sec61α^k04917^* mutant PNS motor neuron pattern (red) shows defasciculation (solid arrows) and lack of Synaptotagmin-1 immunolabeling (green) at motor axon termini (concave arrow) compared to wild type (G, concave arrows). (K) *Sec61α^k04917^* mutant CNS motor neuron pattern (red) shows lack of development of CNS compared to same age wild type embryo (H). CNS axon pathfinding is disrupted along longitudinal connectives (arrow). (L) *Sec61α^k04917^* mutant PNS sensory neuron pattern shows Anterior Fascicle sensory nerve aberrantly crossing hemisegment boundary anteriorly (arrow, compare to (I)). Round sensory neuron cell bodies are disorganized compared to stereotypic wild type pattern. (C, F, I, L) Asterisks indicate lateral chordotonal organs, which are disorganized in both *Sec61α^k04917^* (L) and *Sec61α^SH190^* (F) alleles compared to wild types (C, I).

The *Sec61α* translocon gene has potential relevance to human disease, since it is implicated in polyglutamine-induced neurodegeneration [7],[44]. It is highly conserved, with 91% peptide identity to human Sec61α. *Sec61α* dsRNA-treated cultures were scored both visually and from quantitative analysis as a strong hit that showed defasciculation and excess branching (Figure 3E). To determine whether *Sec61α* hypomorphic mutants have neural outgrowth defects *in vivo*, homozygous *Sec61α^k04917^* [7] and *Sec61α^l(2)SH0190^* [45] mutant embryos were stained with neuronal and glial markers (Figure 4). Both alleles are P element insertions to introns of the *Sec61α* locus [7],[45]. The *Sec61α^k04917^* allele was a stronger hypomorph than the *Sec61α^l(2)SH0190^* allele, yet both showed similar types of nervous system disruption. In the CNS, commissural axon tracts were poorly separated in 33/145 (23%) hemisegments of *Sec61α^l(2)SH0190^* homozygotes (Figure 4B,E, arrows, 145 hemisegments scored) and in 27/105 (27%) of *Sec61α^k04917^* homozygotes, while wild type embryos showed no similar commissural disruptions in 136 hemisegments scored. In the mutants where the CNS commissures were malformed, the midline glia were also aberrantly distributed (Figure 4A,D, arrows). In the stronger *Sec61α^k04917^* allele, the nervous system development was stunted in comparison to wild types. After 22 hours of development, the CNS was structurally mature in all wild type embryos (n = 200), showing well-fasciculated longitudinal axonal tracts and expressing strongly the synaptic marker Synaptotagmin-1 (Figure 4H, green). However after the same time duration in *Sec61α^k04917^* homozygotes, CNS development was representative of earlier stages in all embryos scored, (compare Figure 4H, K) (n = 200).

The PNS of both *Sec61α* alleles showed aberrant motor and sensory axon tracts. The axon bundles appeared defasciculated (Figure 4E, asterisk, Figure 4J, solid arrows), and hemisegments with incorrectly targeted/misbranched axonal projections were identified in 18/145 (12%) of *Sec61α^l(2)SH0190^* homozygotes, and 63/115 (55%) of *Sec61α^k04917^* homozygotes, compared to 2/138 (1%) of wild types. The profiles of the PNS glial processes were abnormal compared to wild types (Figure 4C,F). It has been previously shown that the disruption of embryonic glial development can in turn lead to errors in axon pathfinding [46]. In the *Sec61α* mutants, typical neuronal patterning errors that occur as a result of disrupted glial sheaths were observed, such as the sensory system Anterior Fascicle crossing hemisegment boundaries anteriorly (Figure 4I,L, arrows). These phenotypes could arise because PNS glia contribute to the establishment of the correct positioning and bundling of the peripheral nerves at the CNS/PNS transition zone [47]. We also observed erratic positioning of sensory neurons at the PNS/CNS transition zone in *Sec61α^k04917^* homozygotes 89/127 (70%) (Figure G,J, compare arrowheads) and 57/103 (55%) of *Sec61α^l(2)SH0190^* homozygotes compared to none in wild types (n = 108). Such defects are not likely to be simply due to underlying defects in musculature (Figure S5), since it has been shown that sensory neurons develop rather normally in the absence of mesoderm development [48] and additionally, muscle cells are not known to have an influence on the positioning of CNS/PNS transition zone neuronal exit and entry points.

We also observed a reduction in expression of antigens within the nervous systems of *Sec61α^k04917^* and *Sec61α^l(2)SH0190^* mutant embryos. For example, all heterozygous mutant *Sec61α^l(2)SH0190^* embryos analyzed showed reduced glial mAb 5B12 antigen labeling compared to heterozygotes stained in the same preparation, as well as wild-type embryos. For confocal imaging of embryos in Figure 4, the laser power was increased for the *Sec61α^l(2)SH0190^* specimens to show mAb 5B12 staining. Similarly we observed less Synaptotagmin-1 labeling within *Sec61α^k04917^* mutant embryos (Figure 4G, K, specimens imaged with equivalent laser power in green Synaptotagmin 1 channel). Given the translocon function of the Sec61α protein, it is likely that many neuronal and glial proteins are not being efficiently trafficked within the hypomorphic mutants, leading to disruption of neuronal development. The analysis of mutant embryos extend the RNAi experiments and provide further evidence that the *Sec61α* translocon gene is required for neural development.

### Validation of Ran GTPase in Mouse Neurons

Since a large number of genes identified in the RNAi screen have close homologs to vertebrate genes, we chose to validate another gene in embryonic mouse brains. *Ran GTPase* was a highly conserved gene identified in the full genome screen that showed dramatic effects on neurite outgrowth when knocked down (Figure S2E). *Ran GTPase* is a member of the Ras superfamily that is involved in a variety of cellular process, including nucleo-cytoplasmic transport [49] and mitosis [50]. The *Drosophila* Ran GTPase protein has 87% similarity to mouse and human Ran. Ran binds to the human AR receptor protein, which shows a polyglutamine expansion in Kennedy's Disease, a neurodegenerative disorder [8], but the role of Ran in Kennedy's disease, or in neurodevelopment is not known. In *Drosophila*, *Ran* transcripts are maternally deposited into the embryo. During later embryonic development *Ran* becomes zygotically expressed specifically in the CNS at stage 12 [51], which corresponds to a time of rapid neural cell division and migration.

**Figure 5:**
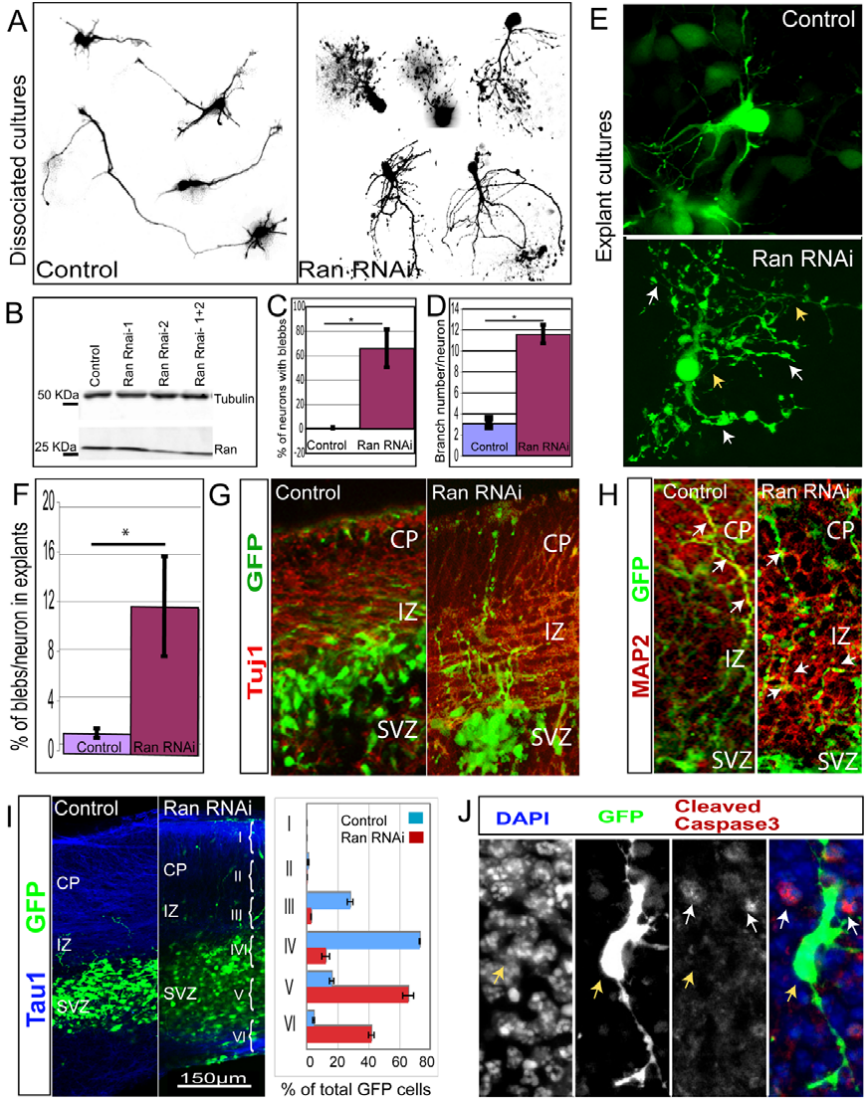
Ran knock down by RNAi results in abnormal neurite morphology in mouse neurons. (A) Cortices from E14 embryos were transfected by electroporation with either a control *GFP* plasmid or *Ran* RNAi contructs, and then dissociated and cultured for 96 hours. Analysis of dissociated neuron morphology shows *Ran* RNAi neurons have increased number of blebs and branches (right panel) compared to control neurons (left panel). (B) *Ran* knockdown in nih-3T3 cells transfected with control GFP vector and *Ran* RNAi constructs. Equal loadings of total protein shown by tubulin signal demonstrates a decrease in the amount of Ran by about 50% in the presence of *Ran* Rnai-2 and when the two constructs are combined. (C) Analysis of the increase in the number of blebs in dissociated neurons. A significant increase in the number of blebs is observed upon *Ran* knockdown (p = 0.02). The average number of neurons with blebs is presented as percentage of the total neuron number (Control = 0.68+/−1.2 SDEV, n = 153; Ran = 66.7+/−15.5 SDEV, n = 36). (D) Analysis of the branching phenotype in dissociated neurons show a very significant increase of branching (p<0.0001) upon *Ran* knockdown (11.56+/−0.88 SEM n = 17) compared to the control neurons (3.07+/−0.41 SEM n = 24). (E) A Z-series reconstruction of a *Ran* RNAi neuron (lower panel) shows an abnormal increase in branch arborization of the processes (yellow arrows) and bleb number (white arrows) compared to the control (upper panel). (F) Analysis of bleb number in explant cultures. The total number of blebs and cell nuclei were counted per section for three independent experiments. The average ratio of bleb per nuclei (shown as a percentage) is significantly increased (p = 0.0007) in the *Ran* RNAi explants (12.6+/−4.2 SEM) compared to the control explants (1.7+/−0.4). (G) *Ran* RNAi neurons labeled with GFP (green) have processes that are immunoreactive for the neuronal marker Tuj1 (in red) (2^nd^ panel) similar to control neurons (1^st^ panel). (H) MAP2 (red), a dendritic marker presents smaller areas of colocalization with GFP (green) in *Ran* RNAi neurons (right panel) compared to control (left panel) white arrows indicate areas of colocalization. (I) Analysis with TAU1 (blue) shows Control (left panel) and *Ran* RNAi (right panel) neurons labeled with GFP (green). Quantitation of GFP cell distribution in electroporated explants shows the percentage of total cells counted per Bin, a representative experiment is shown (error bars are standard error of the mean SEM). Control Bins: II = 0.7+/−0.3; III = 22.9+/−1.5; IV = 59.6+/−0.2; V = 13.1+/−1.2; VI = 3.6+/−0.5. Ran RNAi Bins: II = 0.1+/−0.08; III = 2.3+/−0.06; IV = 9.9+/−2.1; V = 53.6+/−2.9; VI = 34.1+/−1.5. (J) Analysis of cell death in explants with anti-Cleaved Caspase3 (red) immunoreactivity shows that *Ran* RNAi electroporated GFP neurons (yellow arrows) do not colocalize with anti-Cleaved Caspase3 (white arrows).

*Ran* is known to be expressed in the mouse brain at early embryonic stages [52], and is thus a good gene candidate to characterize in mouse brain development using RNAi. We immunolabeled Ran in dissociated cortical neurons and also found high levels of expression in the nuclei of these cells (Figure S4). Furthermore, Ran immunolabeling can be detected in the processes (Figure S4), suggesting a role for *Ran* in neurite outgrowth, as well as in nuclear import. To analyze the role of *Ran* in mouse development, we transfected *Ran* RNAi constructs into the lateral ventricles of the embryonic day 14 (E14) mouse brains using microinjection and electroporation techniques. The transfected cortices were dissected and cultured as explants or dissociated cultures. To test the efficacy of the *Ran* RNAi constructs (1 and 2) in reducing the levels of Ran protein, nih-3T3 cells were transfected with RNAi constructs at 70% transfection efficency. Western blot analysis of total protein from transfected and untransfected cells showed a 64% knockdown of Ran in the presence of *Ran* RNAi construct number 2 (Figure 5B). *Ran* RNAi electroporated neurons showed processes with abnormal blebbing (arrow in Figure 5A right panel) compared to the normal appearing processes in the vector control (Figure 5A left panel). We observed that only 0.7% of control neurons presented blebs while 65.6% of the *Ran* RNAi neurons showed blebs (Figure 5C). The blebbing phenotypes in the mutant compared to wild type was statistically significant (P<0.02). To ensure that the blebs present in *Ran* RNAi neurons were not due to the cell death we analyzed the explants with an apoptosis marker, anti-Cleaved Caspase3. We found that GFP-labeled neurons in the *Ran* RNAi explants did not colocalize with Cleaved Caspase3 (Figure 5J). Thus, the blebbing phenotype was probably due to defects in neurite outgrowth. In addition to the blebbing phenotype, *Ran*-deficient neurons showed an increase in branch arborization (Figure 5A right panel) as compared to the normal branch morphology seen in the control (Figure 5A left panel). The number of branching points per neuron increased significantly (P<0.0001) from 3.2±2.5 in the control to 11.5±3.6 (Figure 5D). We also analyzed the effect of *Ran* deficiency *in vivo* using explant cultures. Analysis of 3D reconstruction of control (Figure 5E top panel) and *Ran* RNAi (Figure 5E bottom panel) showed that the branching and blebbing phenotype is also present in the *in vivo* situation. Quantitation of the number of blebs per nuclei in explant sections showed a very significant (P = 0.0007) increase in *Ran* RNAi deficient explants (Figure 5F). This increased arborization phenotype observed upon knockdown of Ran protein partly resembles the effect of Rac GTPase loss-of-function in mouse and *Drosophila* neurons [53]. Rac GTPases are major regulators of the actin cytoskeleton while the Ran GTPase is a major regulator of the microtubule cytoskeleton. The interplay of both the actin and microtubule cytoskeletons is known to be important for axonal branching [54],[55].

To further characterize the role of Ran in neural development, we immunolabeled sections from *Ran* RNAi-transfected brains with various neuronal markers. In some explants only a few cell bodies of *Ran* RNAi neurons were present in the intermediate zone (IZ) (Figure 5G second panel), compared to the control-transfected cells that were observed to be closer to the IZ and in the cortical plate (CP) (Figure 5G first panel). In other cases, we noted an apparent increase in *Ran* RNAi electroporated cells close to the subventricular and ventricular zone (SVZ/VZ) compared to control explants in TAU1 (axonal marker) immunolabeled explants (Figure 5I). We analyzed the distribution of GFP-positive cells in the explants by dividing the image in Bins I through VI (CP = Bin I–III; IZ = Bin III–IV; SVZ/VZ = Bin IV–VI) (Figure 5I). Three different planes were analyzed per explant. Quantitation of GFP-cell distribution in the explants suggested that upon Ran knockdown (*Ran* RNAi n = 380, 431, 330) the distribution of cells might shift towards the lower areas (Bin IV–VI) compared to the controls (Control n = 284, 461, 476). Immunolabeling for the axonal marker TuJ1 colocalized with processes from *Ran* RNAi transfected cells (Figure 5G right panel) similar to the control transfected cells (Figure 5G left panel). Interestingly, analysis of MAP2 staining showed in some cases higher colocalization of MAP2 and GFP in the control neurons compared to the *Ran* RNAi neurons in the explant cultures (Figure 5H left and right panels). Together, these results suggest an essential and novel role for Ran GTPase primarily in regulation of neurite extension, which in turn could potentially affect neuronal polarity and migration.

## Discussion

We have used an unbiased genetic screening approach to find genes important in neural development by analyzing RNAi-knockdown morphology phenotypes in *Drosophila* primary neurons. We have presented 104 validated candidates from the full-genome screen that represent diverse functional classes such as signaling molecules, ion channels, receptors, enzymes, DNA/RNA associated genes, as well as genes with no predicted functions. The screen also identified numerous genes known to be involved in vesicle and protein trafficking and two of these, *Sec61α* and *Ran GTPase*, showed novel phenotypes when characterized further in *Drosophila* embryonic and mouse cortical explant tissues respectively. Our results show that the primary cell RNAi screening technique is an efficient means of testing functions of genes in neurons, especially in the case of pleiotropic genes where secondary loss of function defects can confound *in vivo* analysis or where maternal RNAs might compensate for loss of zygotic RNA. In the future, since all of the identified genes are evolutionarily conserved, we will conduct *in vivo* analyses in either mice or *Drosophila* using cell biological, physiological, and biochemical techniques. We are also developing new primary neuron RNAi screening assays based on the work presented in this paper to further categorize gene functions in the processes of vesicular trafficking and neurodegeneration. Here we discuss our rationale for using this strategy and how it relates to previous work in neurogenetics.

### RNAi and *Drosophila* in Gene Discovery

RNAi in flies offers unique advantages: physiological assays in living cells are possible to carry out since *Drosophila* grow normally at room temperature, screening can be efficiently carried out on a genome-wide scale, full genome RNAi libraries are openly available for public use, and cell-based RNAi screening can be carried out efficiently without the use of potentially toxic transfection reagents which can significantly increase experimental noise as well as cost [24],[25].

To characterize genes involved in neurodevelopment, the *Drosophila* model is particularly attractive since it has physical and genetic similarities to vertebrates and has particularly well-developed genetic tools [56],[57]. Traditional chemical genetic screens in the fly have provided great insight into major evolutionarily conserved mechanisms of axon guidance by identifying genes such as *slit* and *robo* [58],[59]. Importantly, it is estimated that more than 60% of human disease-associated genes have a closely conserved counterpart in the fly [60],[61]. Recently, *Drosophila* has been used to model human neurological disorders, and to study the genetic pathways associated with diseases such as Parkinson's, Huntington's, and Alzheimer's diseases [62]. For example, analyses of *Drosophila* Huntington's Disease (HD) models have been the first to discover the role of Histone Deacetylases in suppressing HD phenotypes [63], to show disruption of axon trafficking in HD models [42],[64], and to demonstrate that genetic reduction of SUMOylation reduces neurodegeneration of the HD model [65]. There are already some neurological disease models in fly cell culture, such as for Fragile×Mental Retardation [66] and Parkinson's Disease [67], and these could be adapted for use in high-throughput RNAi screening assays such as described here for gaining a better understanding of the disease pathways and for identification of potential therapeutic targets.

### Response of Primary Neurons to Genetic Disruption

While neurobiological analysis of *Drosophila* have most commonly been carried out *in vivo*, assays in primary neural cultures have been conducted for many years and they are known to have numerous features which are particularly advantageous for the use of gene knockdown analysis. In the context of axon outgrowth, it is likely that primary neuronal cultures represent a sensitized system in which the functional mechanisms of molecules can be more easily detected. For example, the addition of cell adhesion proteins Neuroglian and Fasciclin II to primary neuron cultures stimulates axonal outgrowth significantly beyond negative control levels [16], whereas null mutations in *Neuroglian* and *Fasciclin II* cause modest developmental phenotypes. These observations complement our loss of function data, where knockdowns of both genes caused reduced axonal outgrowth. *Neuroglian* was a positive control RNAi for the primary screen (Figure S1) and all subsequent validations, and *Fasciclin II* was a positive hit from the initial primary screen (Table S1). Thus the primary neurons are probably more easily perturbed due to a lack of their *in vivo* permissive growth substrates with which CAMs interact. Since neuronal phenotypes are typically observed only when multiple CAMs are mutated in the same genetic background *in vivo*, it has been suggested that primary neurons are a favorable system to understand the role of CAMs in axon outgrowth [16].

Genetic analyses have also demonstrated close matching of *in vivo* and primary neuron phenotypes. Analysis of the *spalt* gene, which encodes a transcription factor important for neuronal differentiation, shows abnormal axonal branching and Tubulin distribution in primary neurons. *In vivo*, the overall CNS patterning in *spalt* mutants is quite normal, however detailed analysis of *spalt* DiI-labled clones within the embryonic CNS shows phenotypes very similar to the primary cell mutant phenotype [68]. Since fine branching morphologies of neurons are difficult to observe in the densely compacted neuropil CNS, these observations suggest that it can be easier to detect such phenotypes in dissociated cells.

Disruption of axonal trafficking also shows parallels between *in vivo* and primary cell phenotypes. We found that knockdowns of genes known or proposed to be involved in vesicle transport showed increased blebbing in primary neuronal axons as compared to controls. Axonal blebs or swellings are known to occur *in vivo* from disruption of axon transport and are a result of buildup of cargo along the axons [69]. Analysis of axon trafficking of fluorescently labeled cargoes in primary cells could be a convenient screening tool, and could reduce the initial amount of preliminary work needed to identify axon trafficking genes. Assays detecting the localization of subcellular markers for trafficking to specific locations within neurons, such as to neuromuscular junctions, dendrites, and organelles, will help us understand better how the highly polarized structure of the neuron is generated.

Since *Drosophila* primary neural cultures undergo a full range of developmental events starting from neuroblast division to synaptogenesis, the system also provides greater opportunity to find genes necessary for neuronal development and function through screening. In contrast, most *in vivo* screens are designed to assay a specific stage of development. For example the embryonic nervous system can be assayed for the generation of neuronal patterning, but synaptic growth cannot be readily analyzed since physiological activity of neurons is largely absent except at the conclusion of embryogenesis. In addition, primary cultures offer the opportunity to analyze the process of neurodegeneration, which further extends the representative stages of neuronal life that can be observed. One caution is that the distinction between outgrowth versus degenerative phenotypes may not be separable using single time point imaging assays. *Drosophila* primary neuronal axonal processes can retract and lead to shortened axons relative to wild types if vesicle cycling is disrupted [14] or if neurodegeneration occurs [17]. Similarly, RNAi phenotypes of excessive branching in primary neuronal axons could be due to developmental event relating to axogenesis, or could alternately be due to degenerative phenomena and toxicity factors [13],[14]. In the future, automated microscopes with faster image acquisition times will make it more practicable to carry out assays requiring multiple time point analysis.

Inference of specific gene functions from *in vitro* phenotypes also will likely become more accurate when multiple full genome screening neuronal assays have been performed using a variety of cell biological and physiological markers and are cross-compared with bioinformatics tools. We have performed hierarchical clustering analysis on our morphology data set and find that genes known to function in vesicle transport, such as *alphaCOP* and *gammaCOP* show similar morphology feature profiles. Although the pool of genes in our cluster analysis was too small to encompass enough known members of gene classes with closely related functions to identify other important regions of the cluster map, we know from our observations from the primary full genome screen that members of large functional classes such as ribosomal genes show similar knockdown phenotypes in culture (K.Sepp, N. Perrimon, unpublished data) and should in theory cluster together on a hierarchy as well. While we found that the great majority of ribosomal subunits were positively identified in our full-genome screen (K.Sepp, N. Perrimon, unpublished data), for the purposes of bioinformatics this observation does not directly imply that the screen represents near-perfect saturation of the genome. It is likely that certain classes of proteins are more amenable to RNAi than others, as is likely the case for ribosomal subunits. The reasons for this may be protein perdurance, as well as the capability of cells to sometimes function normally with only a fraction of their normal expression of certain genes. Thus bioinformatic analysis of future screens should incorporate an assumption that false negatives do occur. In addition, bioinformatic analyses should include confidence level criteria that relate to whether the screen hits being analyzed were validated through repetitive secondary screening or were simply identified in the initial full-genome screen.

### Summary

In adapting RNAi techniques to assay outgrowth in living primary cells, we were able to perform the first complete genome RNAi screen in neurobiology. We confirmed the RNAi phenotypes of 104 evolutionarily conserved gene candidates that represented a broad range of functional classes. We found that this method is especially helpful in analysis of genes that would be difficult to detect in classical genetic screens due to pleiotropy and other issues. The work presented here lays the foundation for future genetic profiling using a wide variety of assays for specific neural functions as well as physiological assays in living cells.

## Materials and Methods

### Fly Genetics

The fly stock used to generating primary cell cultures for the full genome screen was *w*, *elav^C155^-GAL4*, *UAS-mCD8-GFP/FM6;gcm-GAL4/CyO*. The *gcm-GAL4* line is a product of P-element exchange of the *P[GAL4, w^+^]* with the *P[lacZ, ry^+^]* element in the *rA87* enhancer trap line [70]. Flies were raised in large populations and placed in population cages maintained at 25°C. For cell culture, embryos were collected on molasses plates streaked with paste made from autoclaved yeast mixed with water.

For *in vivo* nervous system analysis, the *Sec61α^l(2)SH0190^* [45] and *Sec61α^k04917^* [71] alleles were analyzed. For unambiguous identification of homozygous mutant embryos, the *Sec61α* alleles were balanced over the *CyO*, *wg^en-lacZ^* chromosome.

### *Drosophila* Cell Culture

Gastrula stage embryos ranging from 6 to 8 hours old were washed from molasses plates with 21°C water, and dechorionated in 50% household bleach in a Nytex-bottomed basket. Embryos were rinsed with sterile distilled water and transferred to Dounce homogenizers containing 100 mL Shields and Sang M3 media (Sigma), and homogenized using 10 pestle strokes. Homogenate was transferred to centrifuge tubes, and debris was pelleted twice with centrifugation at 40 g for 10 min and 5 min. Cells were pelleted by centrifugation at 380 g for 10 min. Cells were resuspended to 2×10^6^ cells/mL using Shields and Sang M3 media (Sigma) supplemented with 10 U/mL penicillin, 10 ug/mL streptomycin, and 200 ng/mL insulin.

### Full Genome RNAi Screen

At the *Drosophila* RNAi Screening Center at Harvard Medical School (http://www.flyrnai.org), dsRNAs were aliquoted in 5 µL volumes into plastic optical-bottomed, black 384-well plates (Corning), with each well containing 250 ng of dsRNAs ranging from approximately 200–500 bp. Specific details on dsRNA generation are previously reported [72]. 10 µL of primary cell preparation (at 2×10^6^ cells/mL) were aliquoted to the dsRNA 384-well plates using a MultiDrop liquid dispenser, and incubated in a humidified chamber for 2 days at 18°C. Following serum-free incubation, cells were supplemented with 30 µL Shields and Sang M3 media (Gibco) containing 5% heat inactivated fetal bovine serum (JRH Biosciences), 10 U/mL penicillin, and 10 ug/mL streptomycin. Cells were incubated an additional 5 days at 18°C in serum-containing media. Following incubation, live, GFP expressing neurons and glia were scored visually, and 512×512 pixel images were collected with a Discovery1 automated widefield microscope (Universal Imaging). Raw images are available upon request.

Control dsRNAs for Rho1, GFP, DIAP-1, and Neuroglian were included on each dsRNA plate. GFP dsRNA served effectively as a negative control, since GFP dsRNA wells were phenotypically indistinguishable from other negative controls such as lacZ dsRNA or water. The GFP dsRNA did not significantly knock down GFP signals in the cells due to the two very strong neuronal and glial promoters simultaneously driving GFP expression, in addition to protein perdurance effects. Except for Rho1, GFP, DIAP-1, and Neuroglian controls on each plate, all wells were scored blind. Importantly, dsRNAs for Rho1, DIAP-1, and Neuroglian were also represented in the dsRNA collection, and were detected as hits when scored blind. The primary screen was carried out on 1–2 independent replicates. For secondary screening, dsRNAs were resysnthesized as reported previously [73]. The secondary screen dsRNAs were aliquoted to 384-well plates in 2 different orientations to avoid plate location artifacts. The secondary validation screens were carried out on 12 independent replicates. For each gene 2 to 3 additional non-overlapping dsRNA amplicons were generated that had no 17–19 bp matches to other genes. Details of the dsRNAs used are publicly available at the DRSC website: www.flyrnai.org.

### Digital Image Analysis

#### Image Feature Extraction of Cell Clusters

From the automated widefield microscopy images, components of the cell phenotypes were quantified. First, neuron cell clusters sizes were determined using the Otsu method (Otsu, 1979), which chooses a global threshold to minimize the intraclass variance of the background and foreground pixels, to convert a raw intensity image to a binary image. The foreground was divided into three categories: small dots (area≤16 pixels), small cell clusters (16 pixels<area≤400 pixels), and large cell clusters (400 pixels<area). Features were computed relating to small dots, small cell clusters, and large cell clusters. A morphological opening was then performed with a disk of radius five to separate cell clusters that are connected by strong axon bundles. This step was followed by a morphological closing operation using the same mask.

#### Image Feature Extraction of Axon Connections

Cell clusters were excluded from the images and we applied a Gabor filter-based approach to compute the axon strength map [74]. The breadth-first search algorithm was applied to the axon strength map and computed all non-overlapped connections between each pair of cell-clusters. The strength of each connection is calculated as the average intensity of pixels that belongs to the connection. Weak connections (strength<0.3) and very long connections (length>300 pixels) were ignored. The ratio between the length of a connection and the Euclidean distance between its two end points was also computed. To keep straight connections, connections with ratios larger than 1.3 were ignored. Finally, the average strength of the connections in an image was computed.

#### Image Feature Extraction of Corners

The corner feature was used to denote the complexity of the axon connections. A set of patterns was defined to search for corners in the binarized axon strength map. For each image, the sum of intensities of the corners in the original raw image was calculated. The sum was then normalized by the square root of the foreground area and denoted as the corner feature of the image.

### Confocal Imaging of Primary Cells and Embryos

For live imaging of primary cells with confocal microscopy, RNAi conditions were scaled up for use with No. 1 1/2 glass bottomed 8-well chamber slides (Nunc). Immediately before imaging, to reduce background fluorescence, the media was removed and replaced with HL6 physiological solution [75]. Cell cultures were imaged using a Leica TCS SP2 AOBS confocal microscope.

For *in vivo* analysis, 12–24 hour embryos raised at 25°C were stained, dissected, and mounted as previously reported [76]. Rabbit anti-HRP::Rhodamine (Jackson ImmunoResearch) was used at a 1∶300 dilution, mouse monoclonal antibodies 5B12 [18], 1D4, 22C10 (Developmental Studies Hypbridoma Bank) were used at 1∶5, rabbit anti-Synaptotagmin-1 was used at 1∶300. Goat anti-mouse Alexa 488, goat anti-mouse Alexa 568, and goat anti-rabbit Alexa 488 (Molecular Probes) were used at 1∶400. Embryo dissections were imaged with a Leica SP2 AOBS confocal microscope. For labeling of primary cells, mAb BP104 (anti-Neuroglian, Developmental Studies Hybridoma Bank) was used at a 1∶10 dilution, and rabbit anti-HRP::Rhodamine (Jackson ImmunoResearch) was used at a 1∶300 dilution. Goat anti-mouse Alexa 488 (Molecular Probes) was used at 1∶400.

### Mouse *in vitro* and *in vivo* Analysis

Cloning of Ran RNAi constructs: Target regions for Ran shRNA hairpin construction were identified using the Broad institute RNAi Consortium database http://www.broad.mit.edu/genome_bio/trc/publicSearchForHairpinsForm.php. Two regions were chosen: (1) CCTTGCTTATTAAAGCCACTA and (2) CGCATCAGATGTTTAAGGATT. shRNA oligos were designed using the Ambion psilencer expression vector insert design tool http://www.ambion.com/techlib/misc/psilencer_converter.html with a loop sequence of TTCAAGAGA and with EcoRI and ApaI restriction site overhangs. The complementary RNAi oligos were annealed at 37°C for 1 hr and ligated overnight at room temperature into an EcoRI/ApaI digested pSil-GFP (gift from Shirin Bonni) [77].

The Psil-GFP plasmid is based on the pSil-1.0 vector and uses a U6 RNA pol III-driven promoter to drive dsRNA and a CMV promoter that drives the GFP. Clones containing the inserts were identified by HindIII and BrSG1 restriction digests, sequenced, and purified for electroporation using an endo-free maxi-prep kit (Qiagen).

Western Blot analysis of Ran knockdown was done by Lipofectamine-2000 transfection of Ran and control constructs into nih-3T3 cells. Cells were cultured for 48, 72 and 96 hour. Cell extracts were made with a micro-homogenizer using 25 mM HEPES pH 7.4, 150 mM NaCl, 0.5 mM EDTA, 0.5% triton, 5 mM MgCl2, phosphatase, protease inhibitors, 2 mM PMSF and 1 mM DTT. Extracts were diluted in protein sample buffer and run on 7.5% PAGE gels using 2 µg of total protein per lane. Western blot protein quantification was done using the Oddyseum Infrared system.

For *in vivo* and *in vitro* analysis, plasmids were transfected by electroporation. Briefly, pregnant Swiss-Webster mice were euthanized at E13.5 and embryos removed. Lateral ventricles were injected using pulled glass microcapillary needles with plasmids in a 0.01% fast green solution (Sigma). Electrodes were placed on either side of the embryo's head and 5×100 ms square pulses at 40v were administered at 950 ms intervals using a BTX830 square-wave pulse generator (Genetronics, Havard Apparatus).

Dissected brains were embedded live in 2% Low melting point agarose in HBSS/pen/strep and sectioned using a Leica vibratome in 250 µm sections that were maintained on organotypic culture membranes for 4 days, fixed in 4% paraformaldehyde and processed for immunofluorescence. Alternatively, cortices were cultured for 24 hours followed by dissociation with papain digestion and trituration using fire polished Pasteur pipettes. Neurons were plated on 12 mm cover slips at high density and examined after 4 days.

Immunofluorescence analysis was done using a Leica Confocal inverted microscope for the explants and an inverted Nikon fluorescence microscope driven by Metamorph software. Immunostaining of dissociated primary neurons was performed as previously described [78]. Ran polyclonal antibody (AbCAM #ab31118) was used at 1 µg/mL. MAP2 monoclonal antibody (Sigma) was used at 1 µg/mL and Tau1 monoclonal antibody (Chemicon) was used at 1 µg/mL. Mouse and Rabbit Alexa 594 secondary antibodies (Molecular Probes) were used at 1∶400. Analysis of the number of branches and blebs was done by counting the number of GFP nuclei and the number of branching points or blebs present in either dissociated neurons or neurons in explants where appropriate.

## Supporting Information

Figure S1 Loss of Neuroglian immunofluorescence in response to RNAi. (A, B) Primary neurons labeled with anti-HRP (A) and anti-Neuroglian (B) shows morphology typical of wild type cultures and similar profile of immuno-fluorescence. C, D) Primary neuron culture treated with Neuroglian dsRNA shows aberrant axon projections when labeled with anti-HRP (C, arrows) and a corresponding loss of anti-Neuroglian immunoreactivity (D).
(1.19 MB TIF)

Figure S2 Design of genome-wide RNAi screen in GFP-labeled primary neural cells. Images acquired on robotic widefield fluorescence microscope, live cell cultures are in 384-well plate format. (A) Wild type control cultures (no dsRNA) show GFP-positive cell clusters interconnected by well-fasciculated axon fascicles. (B) Actin dsRNA treated cultures show smaller cell clusters with weaker connectivity. Axon paths appear abnormally curved compared to wild types. (C) betaTubulin dsRNA treated cells show dramatically reduced axon extension compared to wild types. (D) Schematic of genome-wide RNAi screen. (E) Ran GTPase dsRNA treated cells show excessive branching compared to wild types. Experiments in a1, a2, b1, b2, c1, c2, e1, e2 are generated from the same culture preparation, while experiments in a3, a4, b3, b4, c3, c4, e3, e4 are generated from a separately prepared culture.
(1.63 MB TIF)

Figure S3 Hierarchical cluster of RNAi cell culture image features. Quantified image features from dsRNA-treated cell cultures are are represented values on a blue-red heat map. Wild type control feature values are all white (not shown on hierarchy). Features quantified are: 1) number of small dots (correspond to blebs, debris), 2) total number of corners, 3) corners in major connections between clusters, 4) total number of edges (straight lines correspond to neurites), 5) edges of major connections, 6) area of cell body clusters, 7) median value of cell clusters, 8) number of large cell clusters, 9) number of robust connections between cell clusters, 10) mean straightness of connections between cell clusters, 11) mean connection strength.
(0.55 MB TIF)

Figure S4 Ran immunolabeling of dissociated neuronal culture. Localization of Ran to the nuclei is shown as expected (arrowhead). In addition, Ran is also observed to localize to neuronal processes (arrows).
(0.10 MB TIF)

Figure S5 Embryonic abdominal muscle of wild type and Sec61alpha mutant embryos. Embryos were stained with anti-MHC to show patterning of muscles. (A) The wild type shows stereotyped patterns of muscles in abdominal segments. (B) Sec61alpha k04917 homozygous mutant allele has differentiated muscles and expression of MHC antigen, as does (C), the Sec61alpha l(2)SH190 homozygous mutant allele.
(3.37 MB TIF)

Table S1 Full genome RNAi screen hits previously characterized in neural cells in vivo.
(0.04 MB XLS)

Table S2 Genes showing neural RNAi phenotypes with a single amplicon.
(0.02 MB XLS)
