# Supplementary figures and images for: Identification of Neural Outgrowth Genes using Genome-Wide RNAi
Source: PLoS Genet. 2008 Jul 4;4(7):e1000111. doi: 10.1371/journal.pgen.1000111 (PMC2435276; doi:10.1371/journal.pgen.1000111)

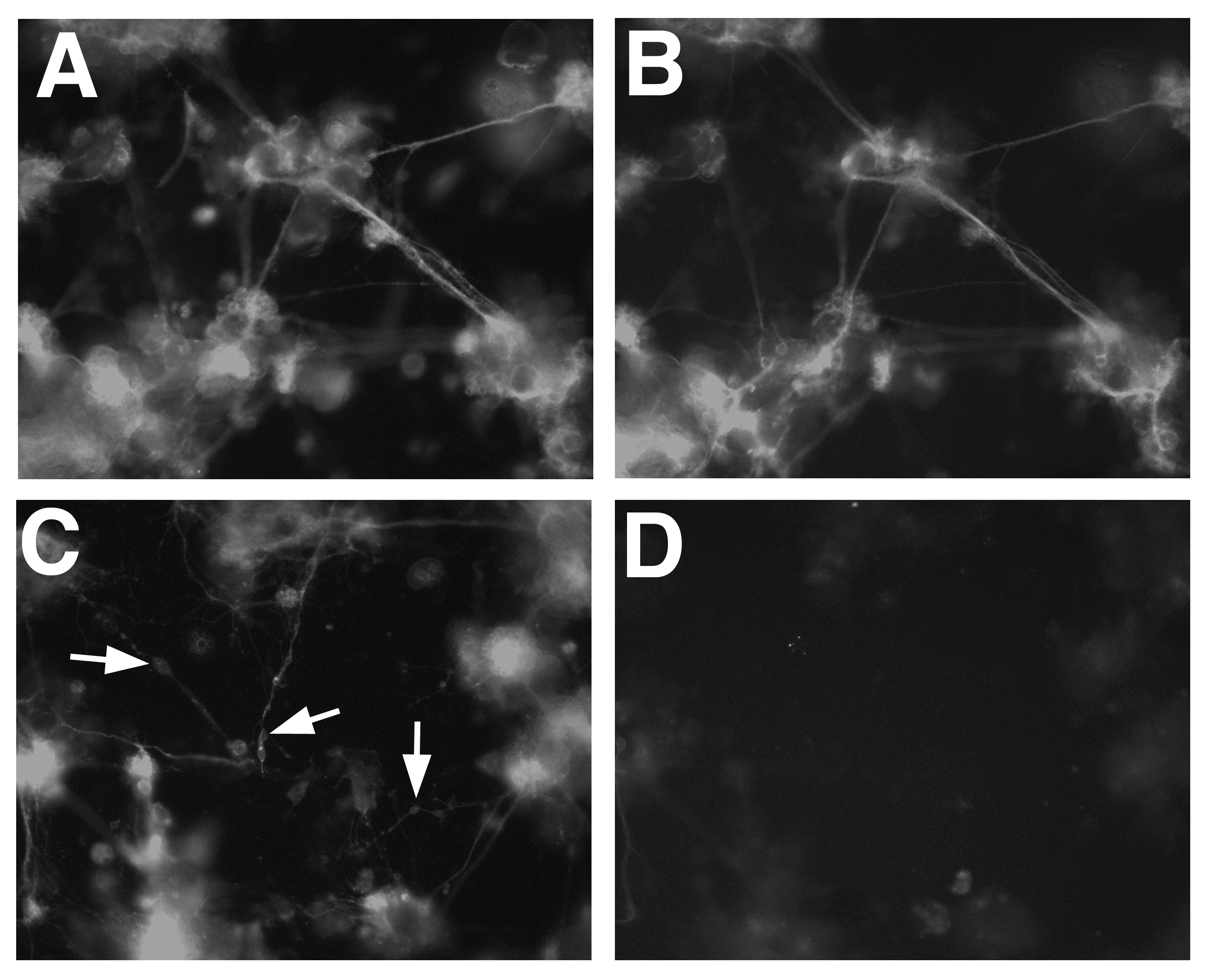

Supplement: Figure S1 — Loss of Neuroglian immunofluorescence in response to RNAi. (A, B) Primary neurons labeled with anti-HRP (A) and anti-Neuroglian (B) shows morphology typical of wild type cultures and similar profile of immuno-fluorescence. C, D) Primary neuron culture treated with Neuroglian dsRNA shows aberrant axon projections when labeled with anti-HRP (C, arrows) and a corresponding loss of anti-Neuroglian immunoreactivity (D). (1.19 MB TIF) [file pgen.1000111.s001.tif]

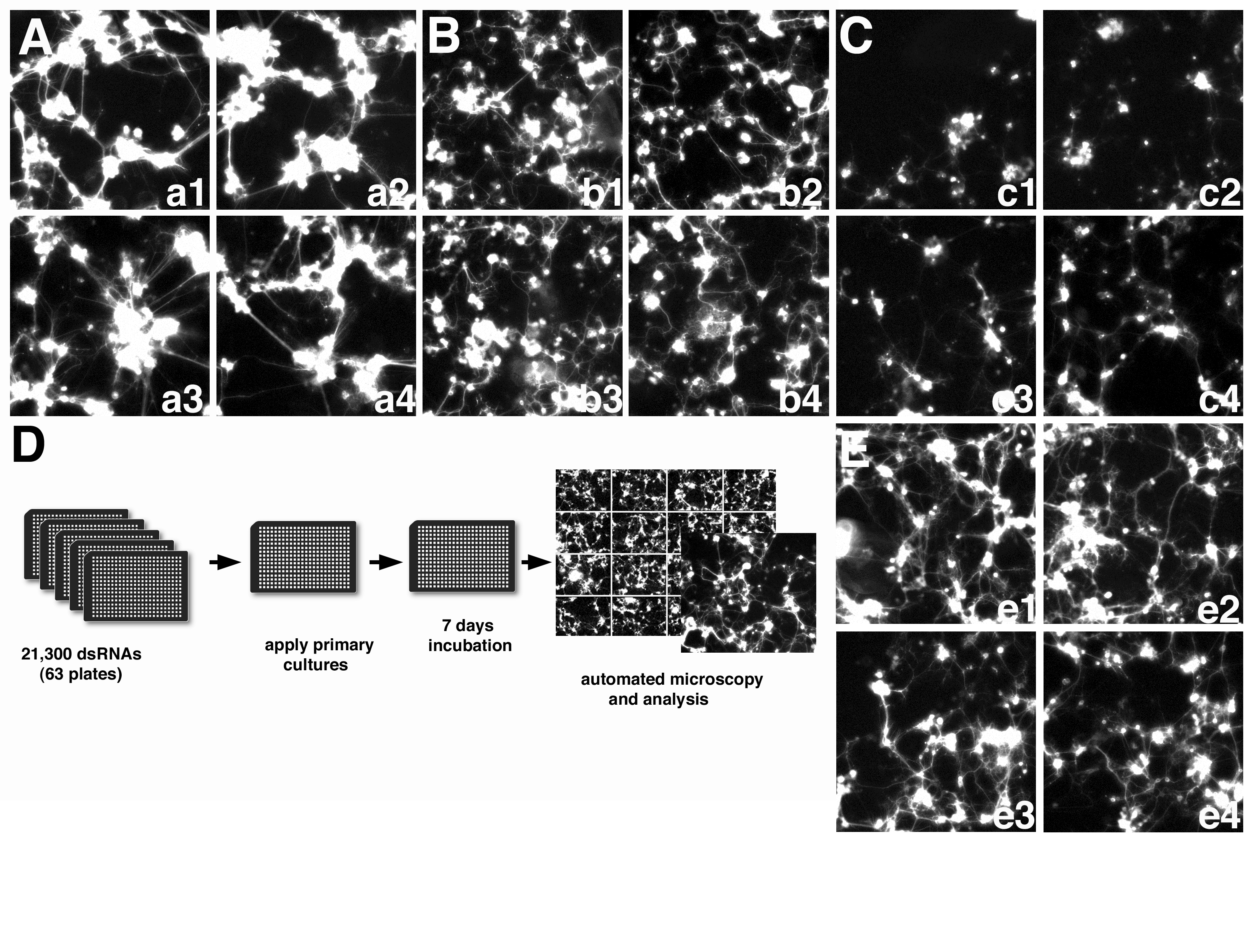

Supplement: Figure S2 — Design of genome-wide RNAi screen in GFP-labeled primary neural cells. Images acquired on robotic widefield fluorescence microscope, live cell cultures are in 384-well plate format. (A) Wild type control cultures (no dsRNA) show GFP-positive cell clusters interconnected by well-fasciculated axon fascicles. (B) Actin dsRNA treated cultures show smaller cell clusters with weaker connectivity. Axon paths appear abnormally curved compared to wild types. (C) betaTubulin dsRNA treated cells show dramatically reduced axon extension compared to wild types. (D) Schematic of genome-wide RNAi screen. (E) Ran GTPase dsRNA treated cells show excessive branching compared to wild types. Experiments in a1, a2, b1, b2, c1, c2, e1, e2 are generated from the same culture preparation, while experiments in a3, a4, b3, b4, c3, c4, e3, e4 are generated from a separately prepared culture. (1.63 MB TIF) [file pgen.1000111.s002.tif]

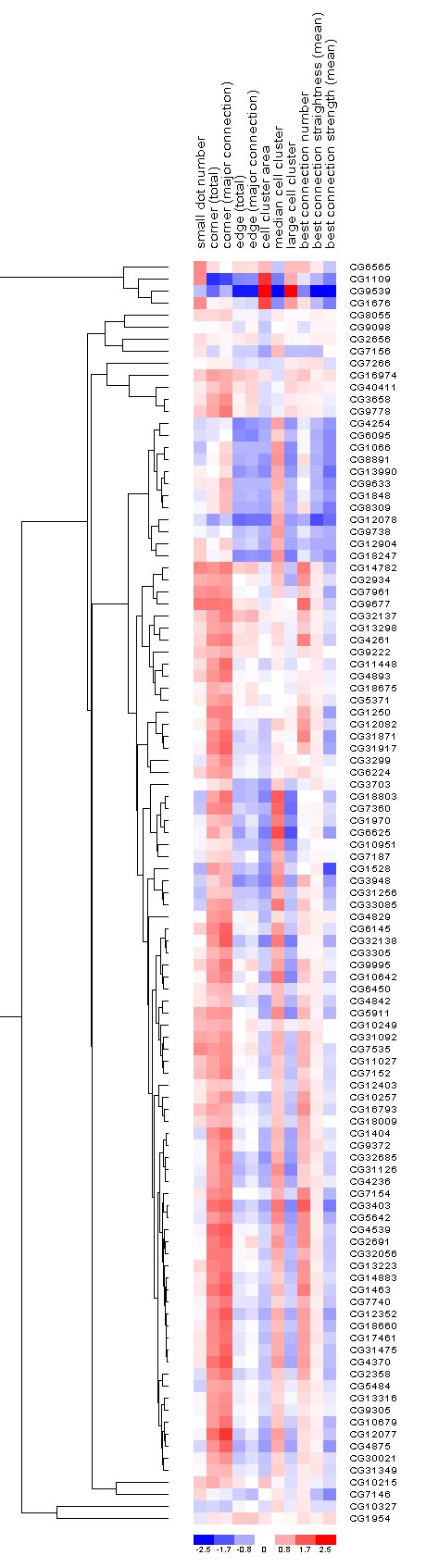

Supplement: Figure S3 — Hierarchical cluster of RNAi cell culture image features. Quantified image features from dsRNA-treated cell cultures are are represented values on a blue-red heat map. Wild type control feature values are all white (not shown on hierarchy). Features quantified are: 1) number of small dots (correspond to blebs, debris), 2) total number of corners, 3) corners in major connections between clusters, 4) total number of edges (straight lines correspond to neurites), 5) edges of major connections, 6) area of cell body clusters, 7) median value of cell clusters, 8) number of large cell clusters, 9) number of robust connections between cell clusters, 10) mean straightness of connections between cell clusters, 11) mean connection strength. (0.55 MB TIF) [file pgen.1000111.s003.tif]

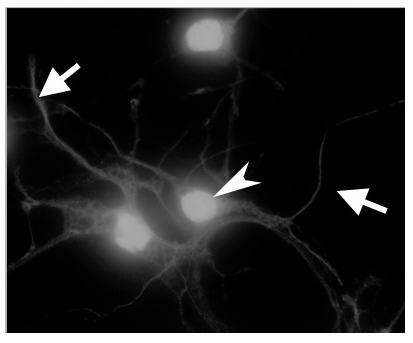

Supplement: Figure S4 — Ran immunolabeling of dissociated neuronal culture. Localization of Ran to the nuclei is shown as expected (arrowhead). In addition, Ran is also observed to localize to neuronal processes (arrows). (0.10 MB TIF) [file pgen.1000111.s004.tif]

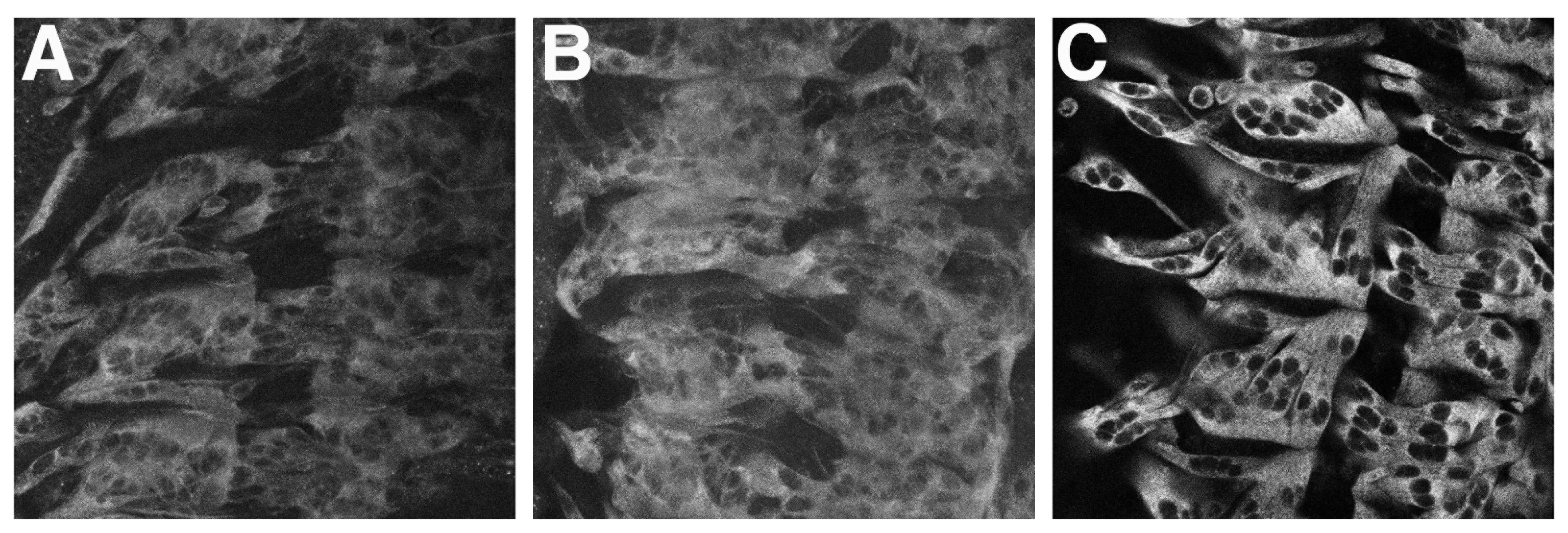

Supplement: Figure S5 — Embryonic abdominal muscle of wild type and Sec61alpha mutant embryos. Embryos were stained with anti-MHC to show patterning of muscles. (A) The wild type shows stereotyped patterns of muscles in abdominal segments. (B) Sec61alpha k04917 homozygous mutant allele has differentiated muscles and expression of MHC antigen, as does (C), the Sec61alpha l(2)SH190 homozygous mutant allele. (3.37 MB TIF) [file pgen.1000111.s005.tif]
